# Effect of Concentrated Apple Extract on Experimental Colitis Induced by Acetic Acid

**Published:** 2017-02-13

**Authors:** Maurício Mercaldi Pastrelo, Carla Caroline Dias Ribeiro, Joselmo Willamys Duarte, Andréa Pitelli Bioago Gollücke, Ricardo Artigiani-Neto, Daniel Araki Ribeiro, Sender Jankiel Miszputen, Celina Tizuko Fujiyama Oshima, Ana Paula Ribeiro Paiotti

**Affiliations:** 1 *Department of Pathology, Universidade Federal de São Paulo, Escola Paulista de Medicina, São Paulo, Brazil.*; 2 *Department of Medicine, Discipline of Gastroenterology, Universidade Federal de São Paulo, Escola Paulista de Medicina, São Paulo, Brazil.*; 3 *Department of Biosciences, Universidade Federal de São Paulo, Santos, Brazil.*

**Keywords:** Experimental colitis, apple extract, antioxidant, iNOS, CuZnSOD

## Abstract

Reactive oxygen and nitrogen species (ROS/RNS) play a crucial role in inflammatory bowel disease (IBD) exacerbating the chronic inflammatory process. Endogenous and diet antioxidants can neutralize these compounds. The apple is widely consumed, with several antioxidant activity compounds. The present study evaluated the effects of concentrated apple extract (CAE) in acetic acid induced colitis. 29 Wistar male rats were randomized into 5 groups. G1–Sham/saline solution, G2–CAE/control, G3–acetic acid/control, G4–curative- CAE treatment and G5–preventive-CAE treatment. Eight days later, the animals were euthanized and the colonic segment resected for macroscopic and histological analysis. Gene expression was evaluated for inducible nitric oxide synthase (iNOS), cyclooxygenase-2 (COX-2), catalase and copper and zinc superoxide dismutase (CuZnSOD) by quantitative real time PCR, while protein expression was assessed for iNOS, COX-2 and 8-hydroxy-20-deoxyguanosine (8-OHdG) via immunohistochemistry. The groups G3, G4 and G5 had weight loss, while G5 had weight increase at the end of the experiment. The treatment with CAE reduced the macroscopic and microscopic injury, decreased iNOS mRNA expression and increased CuZnSOD mRNA expression in animals with induced acetic acid-colitis. The findings of the present study suggest that CAE treatment exerts an antioxidant role by downregulating iNOS and upregulating CuZnSOD.

Inflammatory bowel diseases (IBD) are mainly represented by Crohn’s disease (CD) and ulcerative colitis (UC). They are disorders involving primarily gastrointestinal tract. The pathogenesis is not well understood, but genetic, immunological and environmental factors have been linked to these diseases. It is suggested that epithelial barrier dysfunction may cause the increase intestinal permeability allowing the entrance of lumen antigens leading to recruitment of lymphocytes and macrophages followed by release of soluble cytokines and other inflammatory mediators (1). Subsequent activation of lymphocytes and macrophages causes a self-augmenting cycle of cytokine production, cell recruitment and inflammation. This uncontrolled immune system activation results in a massive production and release of cytokines, inflammatory mediators and reactive oxygen species (ROS) causing intestinal injury (2, 3).

It is suggested that inflammatory response amplification by releasing ROS and inflammatory cytokines and interleukins triggers the pathological responses and symptoms during IBD (2). Thus, activation of inflammatory cascade leads to the release of other mediators such as cyclooxygenase-2 (COX-2) and nitric oxide (NO) (3). NO is an important proinflammatory mediator that is largely produced in three isoforms of NOS: neuronal NOS (NOS1), inducible NOS (iNOS or NOS2) and endothelial NOS (NOS3).

Increased NO levels during colitis leads to an increased peroxynitrite production which ultimately results in oxidation of lipids and proteins, DNA strands breaks, adenosine triphosphate depletion and tissue damage (4). The 8-hydroxy-20-deoxyguanosine (8-OHdG) is a product of oxidative DNA damage formed by the hydroxyl radical, singlet oxygen or by direct photodynamic action. 

Endogenous antioxidant defenses against ROS production act through either enzymatic or non-enzymatic mechanisms. The enzymatic defense system consists mainly of superoxide dismutase (SOD), catalase, glutathione peroxidase (GPx), and glutathione reductase (5). In the enzymatic systems, there are a variety of other non-enzymatic antioxidant molecules such as diet compounds which play key roles in the body defense mecha- nisms (6).

Several specific compounds of fruit and plant extracts are getting more attention as novel agents for therapeutic use. Flavonoids are one of the most abundant natural antioxidants present in human diets. Apple is one of the most cultivated and consumed fruits in the world, rich in antioxidant compounds such as flavonoids, oligomeric procyanidins, dihydrochalcones and hydroxycinnamic acids (7).

Some authors reported the positive effect of polyphenols on experimental colitis. These studies demonstrated the improvement of colonic damage and decrease of inflammatory process (8-11). However, there are few studies that evaluated the beneficial effects of fruits, vegetables and plants without separating their bioactive compounds.

Therefore, the present study aimed to investigate the potential anti-inflammatory and anti-oxidant effect of concentrated apple extract (CAE) on colitis induced by acetic acid (AA). Moreover, to understand these pathways, we investigated the gene and protein expression of iNOS, COX-2, catalase, and copper and zinc superoxide dismutase (CuZnSOD) as well as the protein expression of 8-OHdG.

## Materials and methods

**Animals and experimental design**

Nine– week- old male Wistar rats (200-250 g) were received from Centro de Desenvolvimento de Modelos Experimentais (CEDEME), Universidade Federal de São Paulo, São Paulo, Brazil. Animals were kept under controlled conditions and fed with normal mouse chow and water ad libitum. All experimental procedures of this study were carried out in accordance with the animal ethics committee based on the institutional guidelines.

Rats were divided into 5 groups (n= 5-6 per group): group 1 was kept as normal and received no treatment and group 2 received only the CAE treatment. Group 3, 4 and 5 were subjected to the induction of experimental colitis by intra-colonic application of 1 ml acetic acid (7%). Group 3 was simulated as AA-colitis control; the curative group (G4) was treated with CAE 24 h after AA-colitis induction until the seventh day, and preventive group (G5) was treated with CAE for seven days before and seven days after colitis induction. The groups 2, 4 and 5 received the CAE (1 ml) orally by gavage (equivalent dosage of 4.37 mg polyphenol/day). All rats were euthanized on day 8 following the experiment.

**Concentrated apple extract (CAE)**

CAE was obtained from commercial source Golden Sucos^®^ (Farroupilha, RS, Brazil) at 65º Brix. Total phenols quantification and antioxidant activity of apple extract was performed in a previous study by mass spectrometry (MALDI-MS). The chemical assessment of the apple extract showed a total phenolic concentration of 4.37± 0.06 g GAE/kg and radical scavenging activity of 1.21± 0.03 g VCEAC/kg (5).

**Acetic acid colitis induction**

All animals except group 2 were kept in a 12h fasting with free access to water and were anesthetized with xilazin and ketamine before colitis induction. Acetic acid 7% was administered in a total volume of 1 ml, from the anus via a rubber catheter inserted 7 cm into the colon. Rats from the sham/saline group 1 received 1 ml of saline solution by the same technique. Rats were maintained horizontally for 2 min to prevent leakage.

**Clinical evaluation, macroscopic scoring and histopathological analysis**

During the experiment, changes in body weight and clinical signs were evaluated daily. For macroscopic evaluation, colons were removed and opened longitudinally, cleaned with physiological saline to remove fecal residues. Macroscopic injury was based on the presence or absence of hyperemia, colonic thickening, ulcers, necrosis and adherence to adjacent organs (12). Colons were fixed in 10% buffered formalin (Synth, Diadema, Brazil), embedded in paraffin blocks, and stained with hematoxylin and eosin (H&E) (Merck, Darmstadt, Germany). Histopathological evaluation was performed by a pathologist under a light microscope (Nikon Eclipse E600). Parameters such as crypt destruction, mucosal ulceration, erosion, edema and multifocal inflammatory cell infiltration in submucosa were graded according to Stucchi et al. (13).

**Total RNA preparation**

Frozen colonic tissue was homogenized and total RNA was isolated using cold TRIzol reagent (Invitrogen^®^, Carlsbad, CA, USA) according to the manufacturer’s instructions. Total RNA was determined using a NanoDrop^® ^ND-1000 spectrophotometer (NanoDrop Technologies, Wilmington, DE). RNA samples were treated with DNAse (Invitrogen^®^, Carlsbad, CA, USA) to avoid contamination with genomic DNA. 

**cDNA synthesis and quantitative real time PCR**

cDNA synthesis was performed using the High- Capacity cDNA kit Reverser Transcription (Applied Biosystems^®^, Foster City, CA, USA) according to the manufacturer’s instructions.

TaqMan gene expression assay (Applied Biosystems®, Foster City, CA, USA) was used to quantify mRNA expression. Endogenous genes GAPDH (glyceraldehyde-3-phosphate dehydrogenase) and β-actin were used as internal references. Pre-designed TaqMan gene expression assays were used for studied genes (COX-2: Rn01483828_m1; iNOS: Rn00561646_m1; Catalase: NM_012520.2; CuZnSOD: NM_ 017050.1; GAPDH: Rn01775763_g1; β-actin: Rn00667869_m1). Each reaction was carried out in triplicate in a final volume of 20 µl. The reaction mixture contained 10 µl TaqMan gene expression master mix (Applied Biosystems^® ^Foster City, CA, USA), 1 µl TaqMan gene expression buffer/enzyme (20x) (Applied Biosystems^® ^Foster City, CA, USA), 2 µl cDNA, and 7 µl nuclease-free water. The reaction was carried out in a StepOne Plus thermocycler (Applied Biosystem^®^Foster City, CA, USA) under the following conditions: incubation at 50 ^o^C for 2 min and at 95^ o^C for 10 min, followed by 40 cycles at 95 ^o^C for 15 s and at 60 ^o^C for 1 min. Positive and negative controls were included in each assay.

**Analysis of gene expression data**

To normalize the data for the control and experimental groups, arbitrary units were calculated as: arbitrary unit = 2-ΔΔCT and ΔΔCT = sample ΔCT–control ΔCT, where CT is the threshold cycle.

**Immunohistochemistry**

The slides were deparaffinized in xylene, dehydrated in absolute ethanol and washed with water. Antigen retrieval was performed in citrate buffer, pH 6.0, for 40 min in a steamer. Endogenous peroxidase was blocked by incubation in 3% hydrogen peroxide. Unspecific proteins were blocked by incubation in protein block (Spring Bioscience, Pleasanton, CA) for 15 min at room temperature.

The slides were incubated with goat polyclonal anti-iNOS (Santa Cruz Biotechnology Ltd, Santa Cruz, CA); rabbit polyclonal anti-COX-2 (Santa Cruz Biotechnology Ltd, Santa Cruz, CA) and goat monoclonal anti-8-OHdG (Jaica, Japan) at 4 C overnight. Next, the slides were incubated with the biotinylated secondary antibody of the LSAB kit (Dako, Glostrup, Denmark) for 30 min at room temperature. After washing with phosphate buffer (PBS) the slides were incubated with streptavidin conjugated to peroxidase of the same kit for 30 min at room temperature. The reaction was developed with 3,3’- diaminobenzidine (DAB Liquid, Daki, Glostrup, Denmark) at room temperature. The sections were washed in distilled water, counterstained with Harris’s hematoxylin and mounted in Entellan resin. Evaluation of iNOS and COX-2 reactivity was performed according to a previously described scoring system with staining intensity graded as negative (0), weak (1), moderate (2) and strong (3), and positivity stained area as <10% (0), 10-40% (1), 40-70% (2) and >70% (3) (14). Total scores for grade and area classified as 3 or more were defined as positive and less than 3 as negative. All parameters were evaluated in the cytoplasm of epithelial cells and ulcerated area. The index of 8-OHdG was determined among the total number of positive staining cells per 1000 total nucleus counted on the upper region of the crypt (15).

**Statistical analyzes**

Statistical analysis for the macroscopic, histopathological, mRNA expression of iNOS, COX-2, catalase and CuZnSOD and 8-OHdG positivity was performed by one-way analysis of variance (ANOVA), followed by Tukey’test using Graph Pad Prism (version 4.0). The iNOS and COX-2 positivity were analyzed by Fisher’s exact test using SPSS (version 19). A value of P<0.05 was considered for statistical significance. 

## Results

**Clinical evaluation of orally administered CAE in AA- induced colitis**

**Fig. 1.**
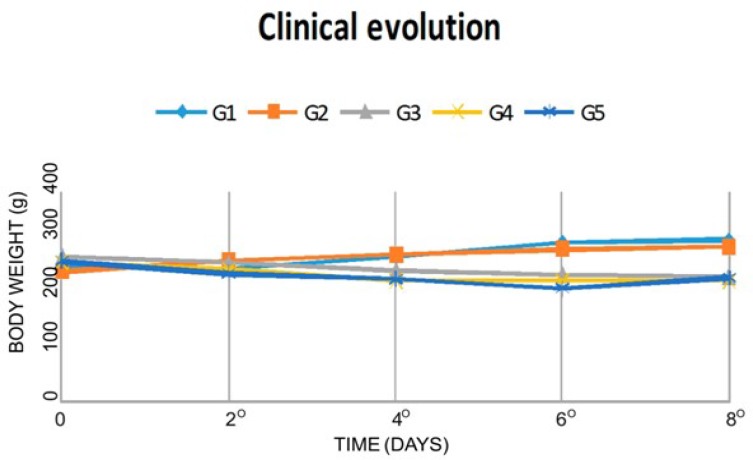
Clinical evolution of the weight in all groups

Initially, clinical signs and corporal/body weight after colitis induction and whether CAE treatments affected these parameters were assessed. Groups 1 and 2 remained without clinical signs and had an increase of the corporal/body weight until the 8th day. On the other hand, groups 3, 4 and 5 presented a decrease in corporal/body weight with statistical significance in comparison with groups 1 and 2 (P<0.05); moreover, they showed loose stools and abdominal distention, being less evident in group 5. However, two days before euthanasia, group 5 had body weight gain, but without statistical significance (Figure 1).

**Effects of CAE on macroscopic evaluation and histopathological analysis of the colonic tissue**

**Fig. 2:**
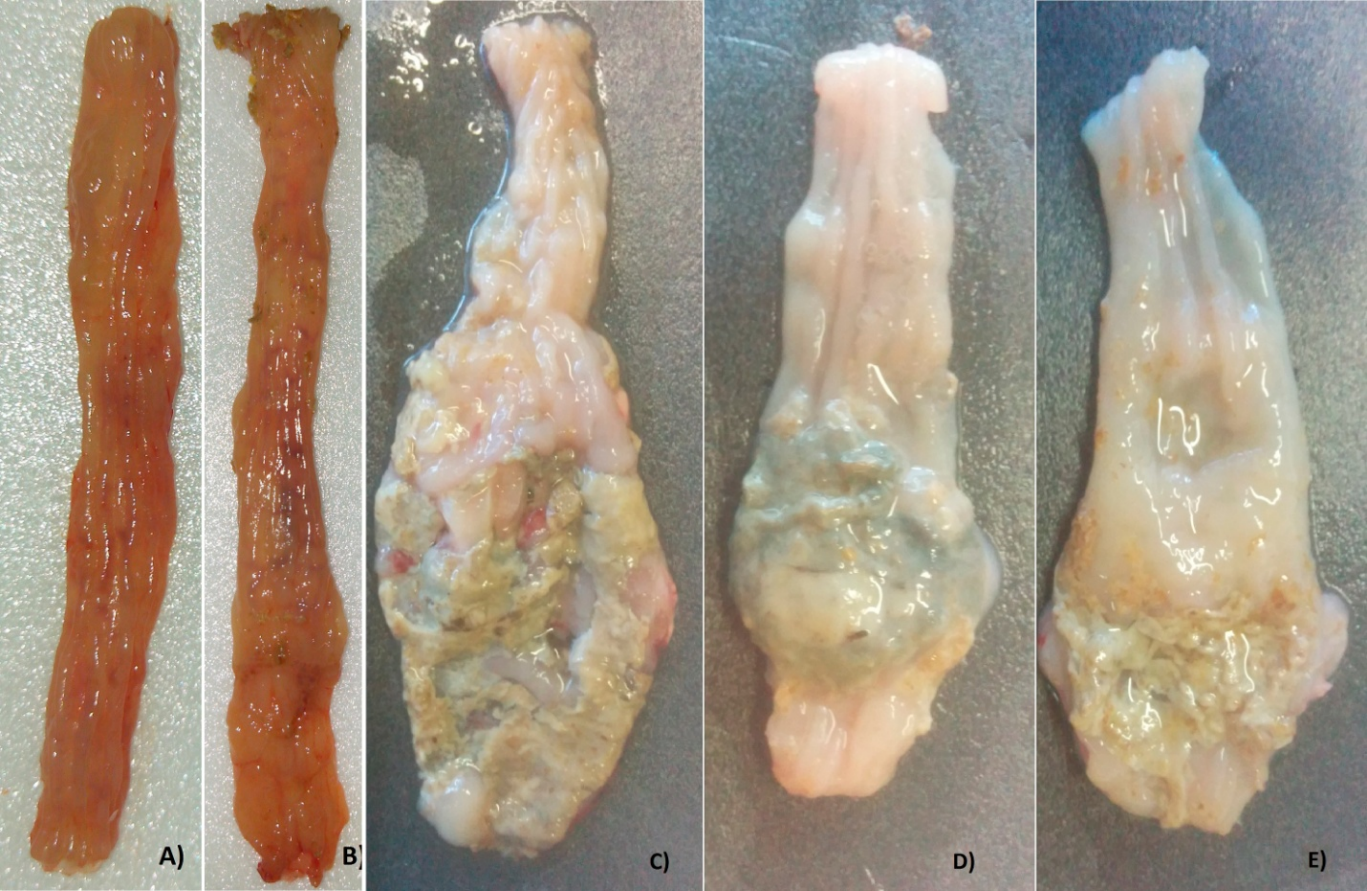
Macroscopic evaluation of the colon. A) G1- sham and B) G2 – CAE control groups: colon without morphological alterations; C) G3 – AA-induced colitis group: severe shortening and thickening of the colon, major ulcers and necrosis; D) G4 – curative CAE treatment and E) G5 – preventive CAE treatment: moderate thickening of the colon and minor ulcerations

**Fig. 3.**
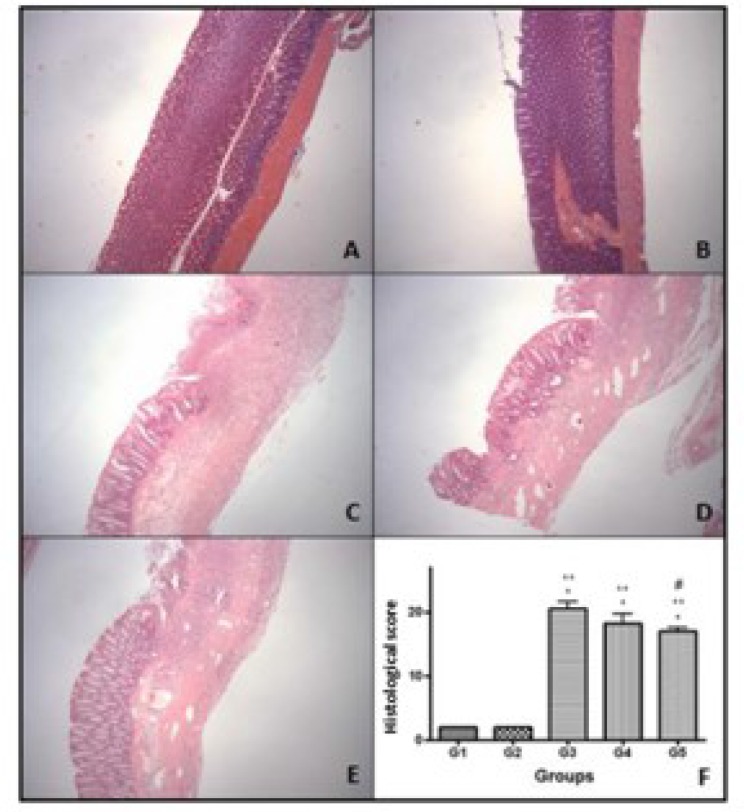
Photomicrograph of the hematoxylin and eosin-stained of colonic sections in the different groups. A) G1 - sham and B) G2 – CAE control groups: normal cellular architecture of the colon; C) G3 – AA-induced colitis group, D) G4 – curative CAE treatment and E) G5 – preventive CAE treatment: intense inflammatory infiltrate, ulceration and distortion of cellular architecture with 40X magnification. F) Score of histopathological analysis (* p<0.01 G3, G4 e G5 vs G1, ** p<0.01 G3, G4 e G5 vs G2, # p<0.05 G5 vs G3) – ANOVA -Tukey

After euthanasia, the macroscopic injury was evaluated with scores ranging from 0 to 12 points. G1 did not score and G2 only showed hyperemia in some rats. G3 showed major damages compared to other groups. The G3 rats presented greater increased colonic thickening, ulcers, necrosis and adherence to adjacent organs. As expected, the AA-induced colitis rats presented greater colonic damage compared to sham and CAE control groups (P<0.001). There was difference between the CAE treated groups and G3 (non-treated group). Treatment with CAE in G4 and G5, significantly decreased the intensity of macroscopic damage (P<0.05). In addition, only 1 rat of G5 presented the adherence to adjacent organs (Table 1; figure 2). Regarding the microscopic analysis, the sham and CAE-control groups (1 and 2, respectively) presented all intact layers. On the other hand, rats exposed to AA showed severe colonic inflammation characterized by crypt destruction, mucosal ulceration, erosion, edema and multifocal inflammatory cell infiltration in submucosa. The mucin depletion was more evident in the AA-induced colitis group (G3). There was no significant difference between groups 3 and 4. Nonetheless, the difference between groups 3 and 5 was observed (P<0.05) (Table 1; figure 3).

**Table 1:** Macroscopic and histological damages

**Groups**	**Macroscopic damages**		**Histological damages**	
	**Mean**	**SE**	**Mean**	**SE**
G1	0.0 *	0.0	2.0*	0.0
G2	1.0 *	0.0	2.0 *	0.0
G3	7.6	1.6	20.5	1.0
G4	4.0**	0.8	18.1	1.5
G5	3.6 **	0.4	17.0 **	0.4

Data are presented as mean± standard errors.

*, **: mean value was significantly different

* P< 0.001

** P< 0.05) when compared with G3 (AA non treated colitis group).

**Effects of CAE on iNOS and COX-2 mRNA expression in colonic tissue**

Increased expressions of iNOS and COX-2 in colonic tissues during ulcerative colitis were related with exacerbation of the inflammatory process. Our data showed high-regulation of these mediators on AA-induced colitis. Nevertheless, we observed a significant decrease of iNOS mRNA expression on curative CAE-treated group (G4) in comparison with non-treated group (P<0.01). In addition, we observed a decrease of COX-2 mRNA expression in the same group but without statistical significance (Table 2).

**Effects of CAE on antioxidant enzyme Catalase and CuznSOD mRNA expression in colonic tissue**

Regarding CuZnSOD mRNA expression, the curative CAE-treated group (G4) showed a significant increase of CuZnSOD (P<0.05) when compared with the non-treated group (G3). On the other hand no difference was observed on catalase mRNA expression.

**Immunolocalization of iNOS and COX-2**

**Fig. 4:**
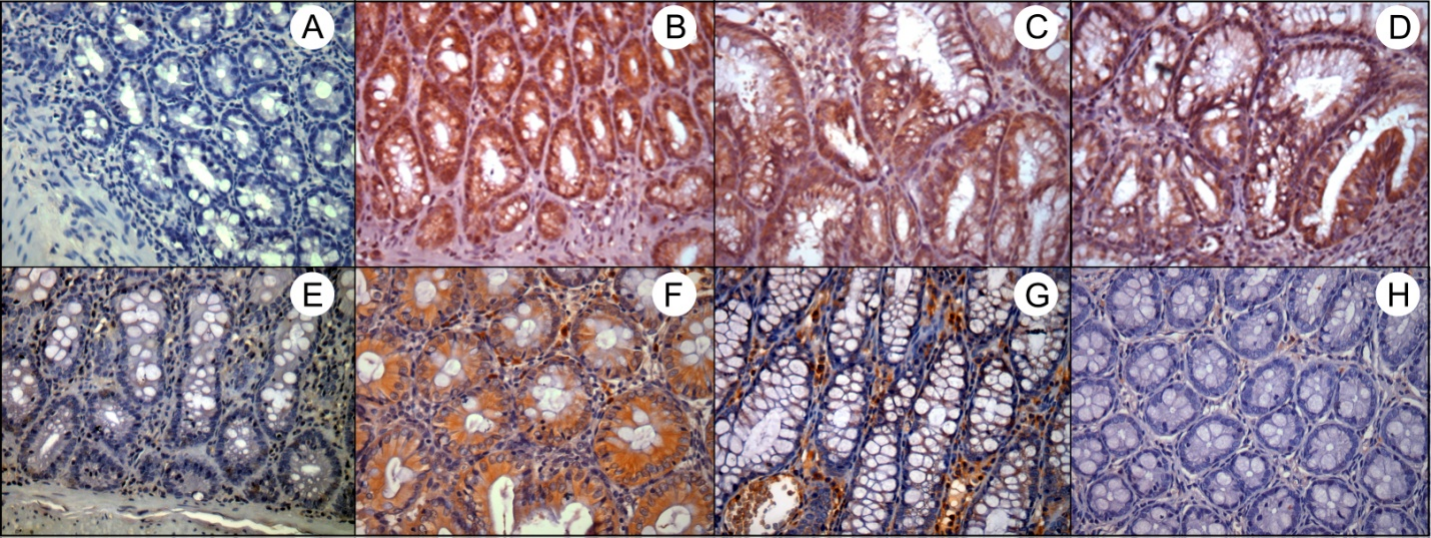
Cytoplasmic immunostaining of iNOS and COX-2 in epithelial cells. A) G1 – Sham control group: negative control; B) G3 – AA-induced colitis control group, C) G4 – curative CAE treatment and D) G5 – preventive CAE treatment: intense staining of iNOS; E) G2 – CAE control group: weak staining of COX-2; F) G3 – AA-induced colitis control group – intense staining of COX-2, G) G4 – curative CAE treatment: moderate staining of COX-2 and H) G5 – preventive CAE treatment: weak staining of COX-2. 400X magnification

**Fig. 5:**
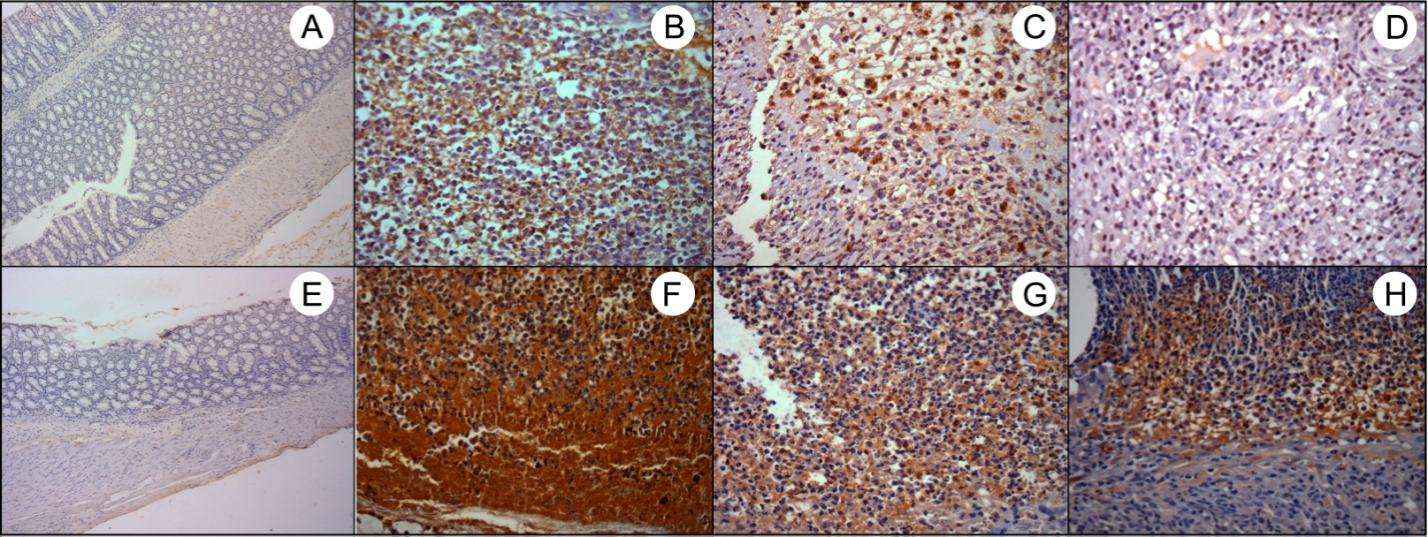
Cytoplasmic immunostaining of iNOS and COX-2 in inflammatory infiltrate cells. A) G1 – Sham control group: as negative control, without ulcerated area; B) G3 – AA-induced colitis control group – intense staining of iNOS, C) G4 – curative CAE treatment: moderate staining of iNOS and D) G5 - preventive CAE treatment: weak staining of iNOS; E) G2 – CAE control group: as negative control, without ulcerated area; F) G3 – AA-induced colitis control group – intense staining of COX-2, G) G4 – curative CAE treatment and H) G5 – preventive CAE treatment: moderate staining of COX-2. 100X and 400X magnification

Regarding iNOS immunolocalization, the CAE-treated groups, especially group 5, showed decrease in ulcerated area but without significant difference. On the contrary, the treatment with CAE did not alter the COX- 2 immunolocalization (Table 3; figures 4 and 5).

**Table 2:** iNOS, COX-2, Catalase and CuZnSOD gene expression.

**Groups**	**Genes**			
	**iNOS**	**COX-2**	**Catalase**	**SOD1**
G1	3.39±1.41	0.81±0.31	3.70±1.05	2.90±0.51
G2	4.15±0.66	0.98±0.18	4.61±1.13	4.12±0.55
G3	10.85±1.63	5.22±1.72	4.31±1.65	3.33±0.80
G4	2.96±0.88^ǂ^	2.19±0.93	4.75±0.35	5.65±0.32^#^
G5	7.15±1.75	4.59±0.64	4.86±0.53	4.10±0.13

Data are presented as mean± standard errors.

ǂ, # : mean value was significantly different (ǂP<0.01/ #P<0.05) when compared with G3 (AA non treated colitis group).

**Table 3:** iNOS, COX-2 and 8-OHdG immunolocalization

**Groups**	**iNOs**		**COX-2**		**8-OHdG** *
	**Epithelium**	**Ulcer**	**Epithelium**	**Ulcer**	
G1	5.6±1.0	0.0±0.0	2.6±0.9	0.0±0.0	214.4±82.43
G2	5.0±0.6	0.0±0.0	2.4±1.1	0.0±0.0	132.6±47.92
G3	5.0±0.6	2.6±0.2	3.6±0.9	6.6±0.6	867.0±32.22
G4	2.2±1.0	1.6±0.2	1.8±1.1	4.0±0.8	776.3±116.5
G5	3.7±1.4	0.8±0.5	2.8±1.3	5.8±1.1	778.5±78.16

Data are presented as mean± standard errors.

* number of positive staining cells per 1000 total nucleus counted on the upper region of the crypt.

**Effects of CAE on 8-OHdG expression in colonic tissue**

**Fig. 6:**
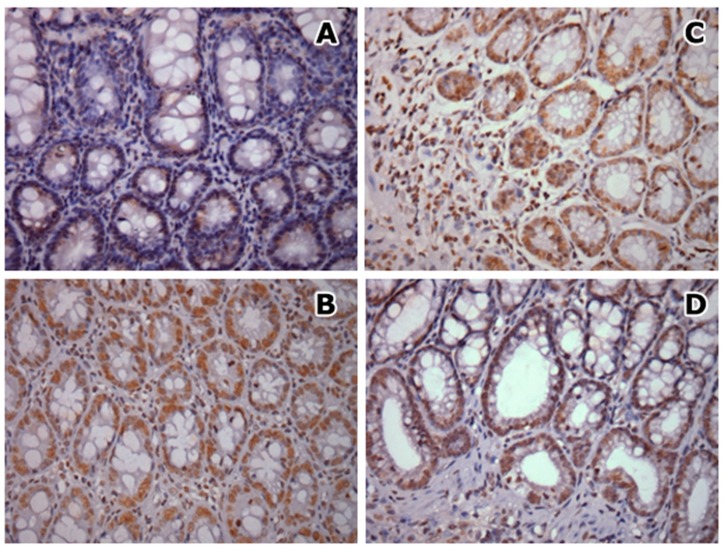
Nuclear immunostaining of 8-OHdG in epithelial cells. A) G2 – CAE control group: weak staining; B) G3 –AA-induced colitis control group; C) G4 – curative CAE treatment and D) G5 - preventive CAE treatment: intense staining. 400X magnification

The sham and CAE-control groups presented low marked nuclei and low labeling index in comparison with AA-induced colitis and CAE-treated groups (P<0.001). Despite, that no significant differences were detected when animals were treated with CAE, either before or after colitis induction. The results are summarized in Table 3; figure 6.

## Discussion

Antioxidants are compounds able to inhibit and retard the substrates oxidation through electron donation, but without becoming free radicals. In this way, the antioxidants might prevent diseases through anti-inflammatory and antioxidants properties. Antioxidants found in plants, vegetables or fruits are beneficial to prevent as well as to serve as complementary treatment in several pathological conditions. In spite of that, in most studies, these natural antioxidant compounds were used in isolation.

Yoshioka et al. in 2008, demonstrated that the oral administration of 1% apple procyanidins before dextran sulfate sodium (DSS)-induced colitis attenuated the mortality rate, decreased body weight loss and degree of colonic injury in C57BL6 mouse (16). Similarly, Skyberg et al. in 2011, showed the same positive results with 1% apple polyphenols treatment (17). In a recent study, the rectal administration of apple extract polyphenols in trinitrobenzenesulfonic acid (TNBS)-induced colitis in Wistar rats for 14 days, decreased the macro and microscopic intestinal damages (18).

Although isolated antioxidants have many benefits in the prevention and treatment of many pathologies, studies have demonstrated the influence of fresh food intake. Epidemiological data show that consumption of fresh fruits is associated with low incidence of cancer (19) and diabetes (20). As studies demonstrated great importance of prevention on disease development, the present study aimed to compare the different times of treatment with CAE in AA-induced colitis. In this way, we evaluated the effects of curative and preventive treatment of CAE on AA-induced colitis.

AA-induced colitis causes colonic epithelial lesions and necrosis associated with neutrophils and macrophage infiltration to the damaged colon indicating inflammatory conditions (21). In the present study, the administration of 7% AA showed a severe colonic inflammation characterized by crypt destruction, ulceration and tissue necrosis associated with inflammatory cell infiltration as well as depletion of colonic mucus. The increase of colon thickening was observed. Although the, rats treated with CAE showed improvement of colonic lesions. Our findings demonstrated that the oral administration of CAE attenuated the disease clinical symptoms and macroscopic and histopathologic damages in the rats colon.

A possible explanation for improvement of AA-induced damage was the preservation of goblet cells in the epithelium of the rats treated with CAE. It is known that goblet cells are responsible for mucin production. Mucins are predominant glycoproteins that form a barrier which protects the bowel against antigens present in the lumen and the adequate intake of short chain fatty acids in the diet is critical to gene expression associated with mucin production (22). Chaim et al. in 2014, evaluated the effect of enema with two different concentrations of sucralfate in rats in colitis model. It was observed that the treatment could reduce inflammation and increase the amount of acid and neutral mucins which demonstrates the beneficial role of mucins in intestinal inflammation (23).

Another factor to be considered is the role of inflammatory cytokines in modulating mucosal immune system where the neutrophils and macrophages are responsible for disrupting epithelial integrity and causing colon injury. The cytokines and TNF-alpha lead to the activation and release of other inflammatory mediators such as iNOS and COX-2, amplifying and perpetuating the inflammatory condition. Thus to complement our findings, we evaluated the mRNA expression of some important mediators and antioxidant enzymes in the proposed model.

In the present study, the treatment with CAE demonstrated a positive effect on iNOS downregulation, especially on the curative CAE treatment (G4). Regarding the preventive CAE-treatment (G5), this downregulation was noted only on immunoreactivity, but without statistical significance. Despite no significant difference, the downregulation of COX-2 was observed on CAE-treated groups. These differences may be due to some limitations of the present study, such as the number of animals and short term period of treatment protocol.

It is important to emphasize that the CAE phenolic compound analysis performed by Ribeiro et al., demonstrated higher levels of chlorogenic acid (CGA) in comparison to other compounds (5). The CGA is formed by esterification of caffeic acid and quinic acid and is one of the most abundant polyphenols in nature (24). This compound is present in several medicinal plants as well as in coffee beans and apples (25, 26). Several studies demonstrated their biological activities in animal models of disease. Yet, the effect of CGA on the inflammatory process in the gastrointestinal tract has not been explored. A study performed by Zatorsky et al. showed the suppression of NF-kappaB activation and reduction of neutrophil infiltration in TNBS-induced colitis by the intracolonic instillation of 20 mg/kg CGA (27).

Another study also reported the modulation of cytokines and inflammatory mediator gene expression and protein levels through the use of apple extract or polyphenols. Ribeiro et al. in 2014, showed the beneficial effect of apple extract by suppressing rat tongue carcinogenesis due to its anti-inflammatory activity and apoptosis induction through the intrinsic mitochondrial pathway, especially by downregulating COX-2 and TNF-alpha expression and increasing cytochrome C (Cyt C) and caspase 3 expression (5). A previous study performed by our research group demonstrated that grape juice treatment decreased the TNF-alpha and iNOS gene and immunoexpression, as well as DNA damage in TNBS-induced colitis models (28).

It is known that NO produced by the inducible isoform iNOS generates free radicals such as peroxynitrite and hydroxyl radical. The damage caused by these radicals to the guanine base generates the 8-OHdG, which in turn, becomes a powerful marker of oxidative stress (29). In the current study, there was no difference of 8-OHdG immunoreactivity on curative or preventive CAE-treated groups. Likely, the short term period of treatment was not able to reverse the damage. Similar results were reported by Oshima et al. in 2015 who found that rats with colorectal-induced cancer, treated with grape juice, did not show a decrease of immunoreactivity for this marker (15).

SOD is a key enzyme which inactivates superoxide ion by transforming it into a more stable metabolite, hydrogen peroxide. This H_2_O_2_ is further converted into water by catalase or Gpx. CuZnSOD, which is localized in the cytoplasm, contains copper and zinc at the catalytic centers and catalyzes O_2_- to H_2_O_2_ (30). Given that ROS are mainly produced inside organelles such as mitochondria (31) and cellular bodies, these organelles are among the first ones to be damaged by ROS. In addition, accumulating oxidative damage could affect the efficiency of mitochondria and further the increase rate of ROS production. The CuZnSOD protects aerobic cells against O_2_ toxicity and lipid peroxidation (32). In the present study, the CAE treatment was able to increase the levels of CuZnSOD mRNA.

Regarding the differences found between gene expression and immunoreactivity data, the present study suggests that the CAE treatment on AA-induced colitis plays an antioxidant role by downregulating iNOS and increasing CuZnSOD gene expression. Meanwhile more investigation is necessary to further understanding mechanisms whereby apple extract acts in this inflammatory process.
